# Seroprevalence of Neutralizing Antibodies to Human Adenovirus Type 4 and 7 in Healthy Populations From Southern China

**DOI:** 10.3389/fmicb.2018.03040

**Published:** 2018-12-10

**Authors:** Xianmiao Ye, Lijun Xiao, Xuehua Zheng, Jinlin Wang, Tao Shu, Ying Feng, Xinglong Liu, Wan Su, Qian Wang, Chufang Li, Ling Chen, Liqiang Feng

**Affiliations:** ^1^State Key Laboratories of Respiratory Diseases, Guangzhou Institutes of Biomedicine and Health, Chinese Academy of Sciences, Guangzhou, China; ^2^University of Chinese Academy of Sciences, Beijing, China; ^3^Center for Disease Control and Prevention of Chenzhou, Chenzhou, China; ^4^The First Affiliated Hospital of Guangzhou Medical University, Guangzhou, China; ^5^School of Biomedical Sciences, Huaqiao University, Quanzhou, China

**Keywords:** human adenovirus type 4, human adenovirus type 7, neutralizing antibody, seroprevalence, Southern China

## Abstract

Human adenoviruses type 4 (HAdV4) and 7 (HAdV7) are two major respiratory pathogens and sporadically cause outbreaks of acute respiratory diseases. The neutralizing antibody (nAb) response to these two adenoviruses in civilian populations, which is important for dissecting previous circulations and predicting potential outbreaks, remains largely unknown. In this study, we generated replication-competent HAdV4 and HAdV7 reporter viruses expressing secreted-alkaline-phosphatase (SEAP), and established neutralization assays to investigate the seroprevalence of pre-existing nAb in healthy volunteers from Hunan Province, southern China. The seropositivity rates are 58.4 and 63.8% for anti-HAdV4 nAb and anti-HAdV7 nAb, respectively. High nAb titers (> 1000) were frequently detected in HAdV4-seropositive individuals, whereas most HAdV7-seropositive volunteers had moderate nAb titers (201–1000). The seropositivity rates of anti-HAdV4 nAb and anti-HAdV7 nAb increase with age, with individuals younger than 20 exhibiting the lowest seropositivity rates. Both seropositivity rates and nAb titers are comparable between different sex groups. Notably, HAdV4-seropositive individuals tend to be HAdV7-seropositive and *vice versa*. Because HAdV4 antisera showed no neutralizing activity to HAdV7 whereas HAdV7 antisera cannot neutralize HAdV4, a subgroup of individuals might be susceptible to infection by HAdV4 and HAdV7 and thus generate nAb to both of them. These results revealed the continuous circulation of HAdV4 and HAdV7 and the lack of protective immunity in more than 35% of people, which emphasized the surveillance of these two HAdVs and the development of prophylactic vaccines.

## Introduction

Human adenoviruses (HAdVs) are non-enveloped, double-stranded DNA viruses belonging to *Adenoviridae*. To date, more than 85 genotypes of HAdVs have been identified and classified into seven species (A-G) on the basis of biological characteristics and genomic homology (Kajan et al., 2017; Hashimoto et al., 2018). HAdVs are common pathogens responsible for a variety of diseases, including acute respiratory disease (ARD), conjunctivitis, gastroenteritis, urinary infection and meningoencephalitis (Ghebremedhin, 2014; Radke and Cook, 2018). HAdV infection in immuno-competent healthy adults is usually asymptomatic (Berciaud et al., 2012). However, in particular populations such as immuno-compromised individuals, infants, and children, HAdV infections may lead to severe and even fatal diseases (Ghebremedhin, 2014; Radke and Cook, 2018). Outbreaks of HAdV infection are frequently reported in crowded populations such as school students and military recruits (Ghebremedhin, 2014).

HAdV4 and HAdV7, two members belonging to species E and B respectively, are major causative pathogens of ARD and acute conjunctivitis (Hong et al., 2001; Erdin et al., 2015). Outbreaks of ARD or conjunctivitis caused by HAdV4 have been reported in the United States, Australia, and Japan (McNeill et al., 2000; Aoki and Tagawa, 2002; Zhang et al., 2016), whereas HAdV7-associated epidemics have also been documented in the United States, China, Japan and the Philippines (Tang et al., 2011; Yamamoto et al., 2014; Scott et al., 2016; Yang et al., 2017). These endemics and epidemics have rendered significant economic and healthy burdens in both military and civilian populations (Hoke and Snyder,  2013). The application of live-oral vaccines against HAdV4 and HAdV7 greatly reduced HAdV-associated ARDs in United States military trainees (Hoke and Snyder,  2013). During the cessation of vaccination between 1999 and 2011, HAdV infections sharply increased to pre-vaccine level (Erdman et al., 2002; Kajon et al., 2007). A retrospective survey revealed that low level of neutralizing antibody (nAb) to HAdV4 and HAdV7 in unimmunized military recruits facilitated the return of HAdV infections (Barraza et al., 1999). Although the new live-oral HAdV4 and HAdV7 vaccines were approved and re-introduced in 2011, vaccination was restricted to military recruits but was not applicable for general populations due to safety concerns (Radin et al., 2014; Choudhry et al., 2016). There is still no adenovirus vaccine for civilian populations worldwide. Besides, the knowledge about the seroprevalence of anti-HAdV4 nAb and anti-HAdV7 nAb in civilian populations is quite limited. We proposed that an investigation of pre-existing nAb could help to understand previous circulations of these two HAdVs and to predict potential outbreaks.

Recently, replication competent and incompetent genetic vectors based on HAdV4 and HAdV7 have been developed (Weaver, 2014; Peng et al., 2016). These vectors have great potency for oral administration (Deal et al., 2013). Several vaccine candidates against human immunodeficiency virus, hepatitis B virus, respiratory syncytial virus, and influenza A H5N1 virus based on HAdV4 or HAdV7 vectors have been undergoing preclinical and clinical trials, and exhibited good safety and immunogenicity (Hung et al., 1990; Hsu et al., 1992; Alexander et al., 2012; Weaver, 2014; Khurana et al., 2015). However, pre-existing nAb to HAdV4 and HAdV7 may limit the efficacy of these vaccines (Dharmapuri et al., 2009; Lanzi et al., 2011). Therefore, investigating the seroprevalence of anti-HAdV4 nAb and anti-HAdV7 nAb can also help to select subpopulations suitable for the application of these vaccines.

In this study, we constructed replication-competent HAdV4 and HAdV7 expressing secreted-alkaline-phosphatase (SEAP), and established convenient neutralization assays based on these reporter viruses. Using these neutralization assays, we investigated the seroprevalence of anti-HAdV4 nAb and anti-HAdV7 nAb in a civilian cohort from Hunan Province, southern China. We also analyzed the distributions of seropositivity rates and nAb titers in different age and sex groups.

## Materials and Methods

### Recombinant HAdV4 and HAdV7 Reporter Viruses

HAdV4 (GenBank NO. KF006344.1) and HAdV7 (GenBank No. HQ659699), two clinical isolates, were kindly provided by Prof. Rong Zhou (Guangzhou Medical University). Recombinant HAdV4 and HAdV7 expressing SEAP were constructed according to previously described methods (Zheng et al., 2017). In brief, viral genomic DNA was extracted by sodium-dodecyl-sulfonate lysis (Sigma-Aldrich, St Louis, MO, United States) followed by phenol-chloroform extraction. The terminal regions of HAdV4 and HAdV7 genome, which were used as homology arms, were amplified by PCR and subcloned into pMD19T-Simple vectors (TaKaRa, Dalian, China) to obtain shuttle plasmids pT-Ad4(L+R) and pT-Ad7(L+R), respectively. A unique restriction site for BamHI was introduced between the two arms, and two restriction sites for AsiSI were introduced at the 5′ terminal of the left arm and the 3′ terminal of the right arm. After digestion with BamHI (Thermo Fisher Scientific, Waltham, MA, United States), linearized pT-Ad4(L+R) and pT-Ad7(L+R) were subjected to homologous recombination with viral genomes in E. coli BJ5183 competent cells (Agilent Technologies, Santa Clara, CA, United States) to obtain genomic plasmids pAd4 and pAd7, respectively. Subsequently, the homology arms for E3 deletion, which contain 500 base pairs (bp) upstream of HAdV4 E3 region, or 520 bp upstream of HAdV7 E3 region, or 710 bp downstream of HAdV4 E3 region, or 660 bp downstream of HAdV7 E3 region, were amplified by PCR. Two shuttle plasmids, p4E3LR and p7E3LR, were constructed by inserting the respective homology arms into pVAX1 plasmids (Thermo Fisher Scientific, Waltham, MA, United States). The ligated homology arms were released from p4E3LR and p7E3LR by digestion with BstZ17I and SgrAI (Thermo Fisher Scientific, Waltham, MA, United States). Meanwhile, pAd4 and pAd7 were linearized by digestion with BclI and EcoRI (Thermo Fisher Scientific, Waltham, MA, United States), respectively. The E3 regions were then deleted by homologous recombination between E3LR fragments and linearized pAd4 and pAd7 to obtain pAd4ΔE3 and pAd7ΔE3, respectively. To construct recombinant HAdV4 and HAdV7 reporter viruses, the coding sequences for SEAP flanked by a CMV promoter and a BGH poly(A) signal were amplified by PCR using pGA1-SEAP as the template (Zheng et al., 2017), and inserted into p4E3LR and p7E3LR to obtain shuttle reporter plasmids pGK43-SEAP and pGK73-SAEP, respectively. Finally, pGK43-SEAP and pGK73-SAEP were linearized by digestion with BstZ17I and SgrAI, whereas genomic plasmids pAd4ΔE3 or pAd7ΔE3 were linearized by digestion with SwaI (Thermo Fisher Scientific, Waltham, MA, United States), and pAd4-SEAP and pAd7-SEAP were constructed by homologous recombination between linearized shuttle reporter plasmids and genomic plasmids. To rescue HAdV4-SEAP and HAdV7-SEAP, pAd4-SEAP and pAd7-SEAP were linearized by digestion with AsiSI (Thermo Fisher Scientific, Waltham, MA, United States) and transfected into HEK293 cells (ATCC). After successfully rescued, HAdV4-SEAP and HAdV7-SEAP were propagated in HEK293 cells and purified using cesium chloride gradient centrifugation. The infectious virus titers were determined as described previously (Aste-Amezaga et al., 2004).

### Human Serum Samples

A total of 1302 healthy blood donors were included in this study. The serum samples were collected in the medical examination center of Center for Disease Control and Prevention of Chenzhou, Hunan Province, China, from January 1, 2017 to July 20, 2017. The volunteers ranged from 7 to 70 years old. The percentages of male and female were 54.1 and 45.9%, respectively. The demographic information of serum samples was summarized in Supplementary Table S1. Fresh blood samples were collected into serum tubes and let stand for 30 min at room temperature. Serum samples were then isolated by centrifugation at 3000 rpm for 10 min. 1302 serum samples were obtained, heat-inactivated for 90 min at 56°C and preserved at -80°C. The research with human samples was approved by the Ethics Committee of Guangzhou Institutes of Biomedical and Health (GIBH). Informed consents from each volunteer were signed.

### Mouse Antisera

Six-week-old female BALB/c mice were purchased from Beijing Vital River Laboratory Animal Technology Co., Ltd., and housed in the Animal Experimental Center of GIBH. Animal experiments were approved by Institutional Animal Care and Use Committee of GIBH (IACUC, No. 2015014). HAdV4 antisera and HAdV7 antisera were prepared according to previously described methods (Feng et al., 2018). In brief, HAdV4 and HAdV7 were treated with 0.2% of β-propiolactone (BPL) (Tokyo Chemical Industry, Japan) overnight at 4°C followed by incubation for 2 h at 37°C. In this condition, HAdVs were completely inactivated. Mice were then intramuscularly inoculated with inactivated HAdV4 or HAdV7 at 1.0 × 10^9^ viral particles (vp) per mouse. At 4 weeks after the first immunization, the mice were boosted with similar formulations. At 2 weeks post the final immunization, the mice were bled and euthanized. Serum samples were then collected, inactivated and preserved at -80°C.

### HAdV4 and HAdV7 Neutralization Assays

The nAb titers against HAdV4 and HAdV7 were determined using neutralization assays based on HAdV4-SEAP and HAdV7-SEAP, respectively. In brief, HEK293 cells were seeded into 96-well plates at 3 × 10^4^ cells per well. One day later, HAdV4-SEAP or HAdV7-SEAP at 4 × 10^6^ vp per well (∼100 vp/cell) were incubated for 1 h at 37°C either alone (virus infection alone) or together with serial dilutions of human sera or mouse antisera. Subsequently, each sample was added to the 96-well plates and incubated for 24 h at 37°C. Finally, the supernatants were harvested and the SEAP activity was assessed using the chemiluminescent substrate CSPD according to the manufacturer’s protocols (Thermo Fisher Scientific, Waltham, MA, United States). The relative light units (RLU) were recorded using a luminometer (MlX Microtiter, Dynex Technologies, Inc., United States) and the titers were calculated as the dilutions that inhibited 50% of RLU values.

### Statistical Analysis

Comparisons of the seropositivity rates in different groups were conducted using Chi-square test or Chi-square test for trend. Comparisons of the nAb titers among groups were performed using the Mann-Whitney test or the Kruskal-Wallis test. SPSS version 13.0 (SPSS Inc., Chicago, IL) was used for statistical analyses, and *p*-values less than 0.05 were considered statistically significant. The graphs were generated with GraphPad Prism version 6 (GraphPad Software, La Jolla, CA, United States).

## Results

### Establishment of Neutralization Assays With HAdV4-SEAP and HAdV7-SEAP

**FIGURE 1:**
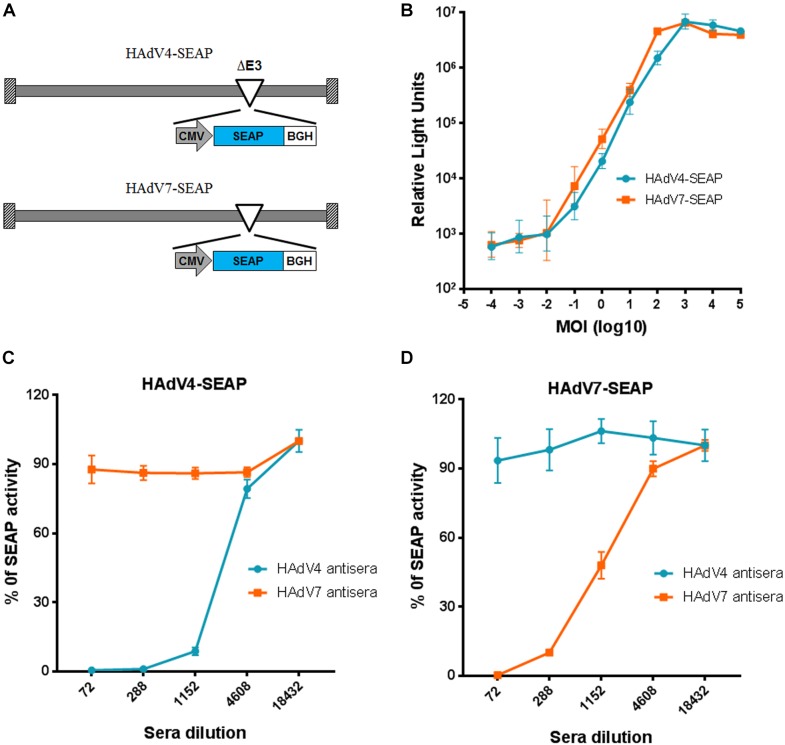
Establishment of neutralization assays based on HAdV4-SEAP and HAdV7-SEAP. **(A)** The schematic diagram of HAdV4-SEAP and HAdV7-SEAP. **(B)** Detection of SEAP activity in the culture supernatants of HAdV4-SEAP or HAdV7-SEAP infected cells. HEK293 cells were infected with HAdV4-SEAP or HAdV7-SEAP at serially increasing doses. At 24 h after infection, the culture supernatants were collected and the SEAP activity was assessed. Shown are the representative data of three independent experiments. **(C,D)** Neutralization curves of HAdV4 antisera and HAdV7 antisera to HAdV4-SEAP **(C)** and HAdV7-SEAP **(D)**. Mouse antisera were serially diluted and incubated with HAdV4-SEAP **(C)** or HAdV7-SEAP **(D)**. After incubation for 1 h, the mixtures were added to HEK293 cells. One day later, the SEAP activity in the culture supernatants were examined. Cells infected with HAdV4-SEAP or HAdV7-SEAP in the absence of antisera were used as viral infection alone controls. Shown are the percentages of SEAP activity in the presence of antisera versus viral infection alone. The data were representative of two independent experiments. Error bars stand for mean ± standard deviation (SD).

To establish a new neutralization assay alternative for traditional cytopathic effect based neutralization assays, we constructed recombinant HAdV4 and HAdV7 reporter viruses expressing SEAP (Figure 1A). HAdV4-SEAP and HAdV7-SEAP were successfully rescued and propagated. The SEAP activity was linearly correlated with the infection dosage of HAdV4-SEAP and HAdV7-SEAP from 0.01 vp/cell to 100 vp/cell (Figure 1B). We chose an infection dosage of 100 vp/cell for assessing the titers of nAb in the serum samples.

We established the neutralization assays using mice antisera generated by immunizing BALB/c mice with inactivated HAdV4 or HAdV7 viral particles. The neutralization curve of antisera to HAdV4-SEAP and HAdV7-SEAP were shown (Figures 1C,D). SEAP activity in the culture supernatants sharply decreased at certain dilutions of serum samples, reflecting the neutralization of antisera to HAdV4 and HAdV7. Notably, SEAP expression in cells infected with HAdV4-SEAP was only suppressed by HAdV4 antisera but not significantly influenced by HAdV7 antisera, whereas SEAP expression in HAdV7-SEAP infected cells was only inhibited by HAdV7 antisera but not HAdV4 antisera. This result suggested that HAdV4 specific nAb have minimal, if any, cross-neutralizing activity to HAdV7, and *vice versa*.

### Overall Seroprevalence of Anti-HAdV4 nAb and Anti-HAdV7 nAb in Healthy Populations From Hunan Province, Southern China

**FIGURE 2:**
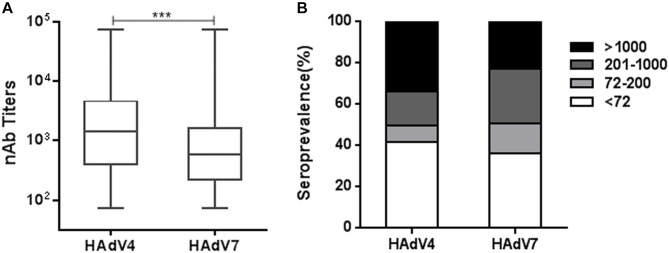
The overall seroprevalence of anti-HAdV4 nAb and anti-HAdV7 nAb. **(A)** The nAb titers against HAdV4 and HAdV7 in seropositive individuals. The data was analyzed with the Mann-Whitney test. ^∗∗∗^*P* < 0.001. **(B)** The distributions of anti-HAdV4 nAb and anti-HAdV7 nAb titers. nAb titers were classified into four subgroups: negative, < 72; low, 72–200; moderate, 201–1000; and high, > 1000. The percentages of individuals with negative, low, moderate, and high nAb titers were shown.

Using reporter virus based neutralization assays, we investigated the seroprevalence of anti-HAdV4 nAb and anti-HAdV7 nAb in a cohort of 1302 healthy volunteers from Human Province, China (Supplementary Table S1). The overall seropositivity rate of anti-HAdV4 nAb was 58.4% (95% Confidence Interval, CI, 55.7–61.1%), which was significantly lower than that of anti-HAdV7 nAb (63.8%, 95% CI 61.2–66.4%) (Chi-square test, *P* < 0.01; Supplementary Tables S2, S3). In the whole cohort, HAdV4-seropositive individuals had significantly higher nAb titers than HAdV7-seropositive ones (Mann-Whitney test, *P* < 0.001, Figure 2A). According to nAb titers, the serum samples were categorized into four subgroups: negative, < 72; low, 72–200; moderate, 201–1000; and high, > 1000. The distributions of different nAb titers were analyzed (Figure 2B). 34.0% of serum samples (95% CI 32.6–38.0%) contained high nAb titers to HAdV4, whereas only 22.9% (95% CI 21.9–26.9%) of serum samples had high nAb titers to HAdV7 (Chi-square test, *P* < 0.001). Significantly more serum samples contained moderate nAb titers to HAdV7 (26.8%; 95% CI 24.4–29.2%) than to HAdV4 (16.3%; 95% CI 14.3–18.3%) (Chi-square test, *P* < 0.001, Figure 2B). These results suggested that although the seropositivity rate of anti-HAdV4 nAb was lower than that of anti-HAdV7 nAb, HAdV4-seropositive individuals tended to have higher nAb titers in comparison with HAdV7-seropositive individuals.

### Seropositivity Rates but Not Titers of Anti-HAdV4 nAb and Anti-HAdV7 nAb Increased With Age

**FIGURE 3:**
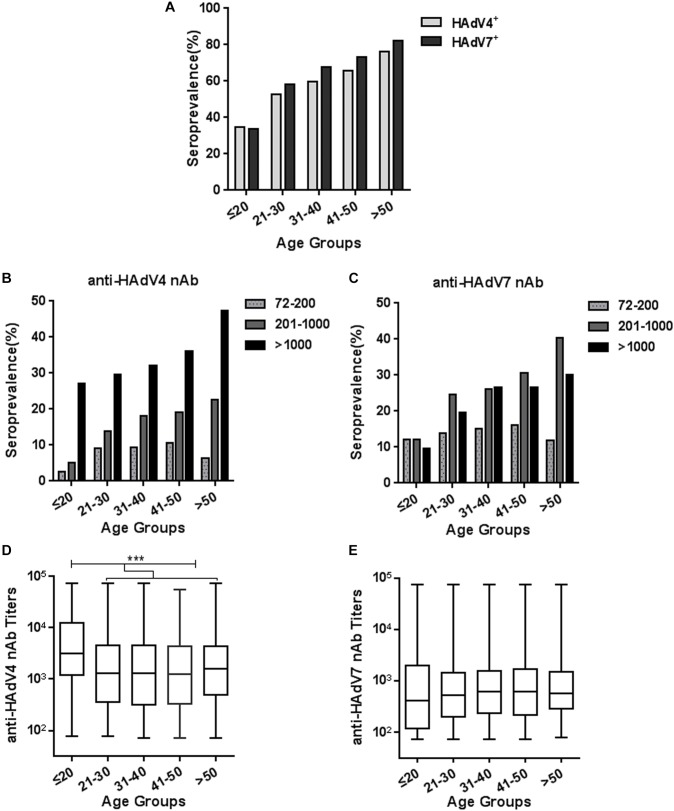
Seroprevalence and titer distribution of anti-HAdV4 nAb and anti-HAdV7 nAb in different age groups. **(A)** The seropositivity rates of anti-HAdV4 nAb and anti-HAdV7 nAb in different age groups. The data were analyzed with Chi-square test for trend. **(B,C)** The distributions of the low (72–200), moderate (201–1000), and high (> 1000) titers of anti-HAdV4 nAb **(B)** and anti-HAdV7 nAb **(C)** in different age groups. **(D,E)** The titers of nAb to HAdV4 **(D)** and HAdV7 **(E)** in different age groups. The data was analyzed with the Kruskal-Wallis test. ^∗∗∗^*P* < 0.001.

We analyzed the trends of seroprevalence in different age groups. The overall seropositivity rates of anti-HAdV4 nAb and anti-HAdV7 nAb increased with age (Chi-square test for trend, HAdV4: *P* < 0.001; HAdV7: *P* < 0.001; Figure 3A). The frequency of serum samples with moderate and high anti-HAdV4 nAb and anti-HAdV7 nAb titers also progressively increased with age (Figures 3B,C). However, the frequency of serum samples with low anti-HAdV4 nAb and anti-HAdV7 nAb titers increased with age before 50 but decreased thereafter (Figures 3B,C). Notably, in each age group, the percentage of individuals with high anti-HAdV4 nAb titers was higher than that with moderate and low anti-HAdV4 nAb titers (Figure 3B), whereas individuals with moderate anti-HAdV7 nAb titers dominated in HAdV7-seropositive ones (Figure 3C). The titers of anti-HAdV4 nAb was higher in the age group younger than 20 than the other age groups (Kruskal-Wallis test, *P* < 0.001; Figure 3D). However, no difference was observed for the titers of anti-HAdV7 nAbs among age groups (Kruskal-Wallis test, *P* = 0.399; Figure 3E). These results revealed a different distribution of seropositivity rates as well as nAb titers for HAdV4 and HAdV7, and implied that individuals younger than 20 may be more susceptible to HAdV4 and HAdV7 infection than older individuals because of relatively lower nAb seropositivity rates.

### Seropositivity Rates and Titers of Anti-HAdV4 nAb and Anti-HAdV7 nAb Were Not Affected by Sex

**FIGURE 4:**
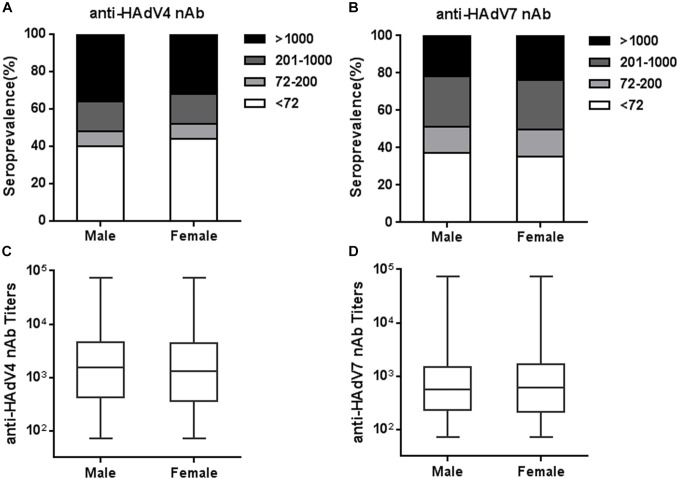
Seroprevalence and titer distribution of anti-HAdV4 nAb and anti-HAdV7 nAb in sex groups. **(A,B)** The distributions of anti-HAdV4 nAb **(A)** and anti-HAdV7 nAb **(B)** titers in sex groups. **(C,D)** The titers of nAb to HAdV4 **(C)** and HAdV7 **(D)** in seropositive males and females. The data were analyzed using the Mann-Whitney test.

We next analyzed the seroprevalence and titer distributions of anti-HAdV4 nAb and anti-HAdV7 nAb in sex groups. The seropositivity rates of anti-HAdV4 nAb in males was slightly higher than that in females, but this difference did not reach statistical significance (Figure 4A). Similarly, no significant differences were observed for the seroprevalence of anti-HAdV7 nAb between males and females (Figure 4B). The titers of anti-HAdV4 nAb and anti-HAdV7 nAb in seropositive males were also comparable to those in seronegative females (Figures 4C,D). Therefore, the seroprevalence of anti-HAdV4 nAb and anti-HAdV7 nAb were associated with age, but not with sex.

### Analysis of the Seroprevalence and nAb Titers in Double-Positive and Single-Positive Individuals

**FIGURE 5:**
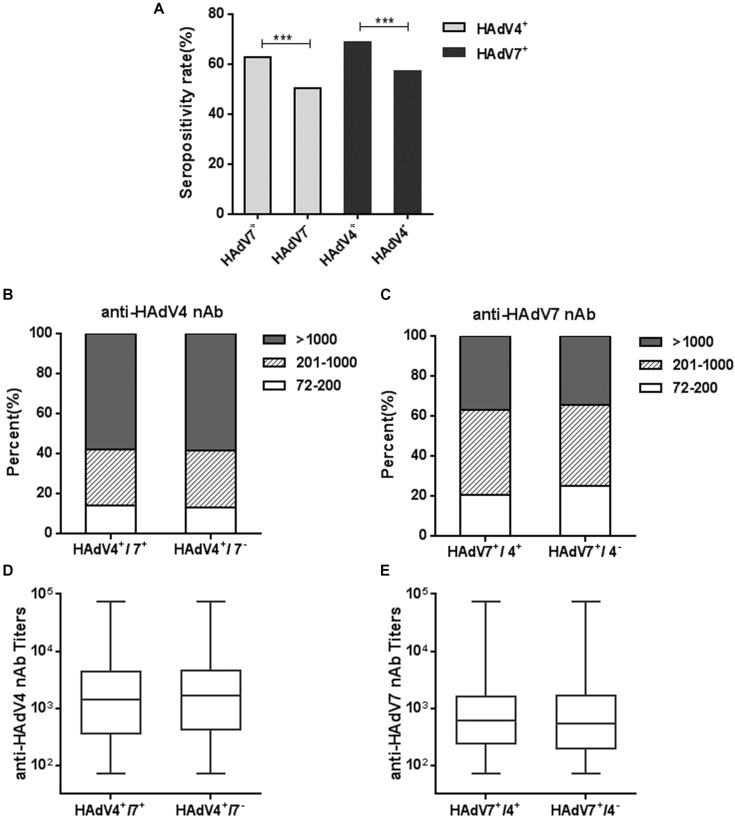
Seropositivity rates and titers of anti-HAdV4 nAb and anti-HAdV7 nAbs in single- and double-positive samples. **(A)** The seropositivity rates of anti-HAdV4 nAb and anti-HAdV7 nAb in single-positive and double-positive serum samples. The data were analyzed with Chi-square test. ^∗∗∗^*P* < 0.001. **(B,C)** The distributions of anti-HAdV4 nAb **(B)** and anti-HAdV7 nAb **(C)** titers in single-positive and double-positive serum samples. **(D,E)** Overall titers of anti-HAdV4 nAb **(D)** and anti-HAdV7 nAb **(E)** in single-positive and double-positive serum samples. The data were analyzed using the Mann-Whitney test.

We also analyzed the seropositivity rates of anti-HAdV4 nAb in HAdV7-seropositive or -seronegative individuals. Significantly more HAdV4-seropositive samples were detected in HAdV7-seropositive individuals than in HAdV7-seronegative ones (Chi-square test, *P* < 0.001; Figure 5A). The percentages of sera with low, moderate and high anti-HAdV4 nAb titers in HAdV7-seropositive individuals were similar to those in HAdV7-seronegative ones (Figure 5B). Similarly, the seropositivity rate of anti-HAdV7 nAb in HAdV4-seropositive individuals was significantly higher than that in HAdV4-seronegative ones (chi-square test, *P* < 0.001; Figure 5A). The percentages of sera with low, moderate and high anti-HAdV7 nAb titers in HAdV4-seropositive individuals were also comparable to those in HAdV4-seronegative ones (Figure 5C). Moreover, the titers of anti-HAdV4 nAb and anti-HAdV7 nAb in double-positive sera were comparable to that in single-positive sera (Figures 5D,E). These results revealed that HAdV7-seropositive individuals tend to be HAdV4-seropositive, and *vice versa*, but the nAb titers to these two HAdVs were comparable in single- and double-positive individuals.

## Discussion

HAdV4 and HAdV7 represent two major respiratory pathogens in civilian populations and military trainees worldwide (Top, 1975; Tang et al., 2011; Yamamoto et al., 2014). However, surveillances of nAb to these two HAdVs in large civilian cohorts are extremely rare, possibly due to the lack of convenient neutralization assays. Here, we developed neutralization assays using HAdV4-SEAP and HAdV7-SEAP (Figure 1). These assays have been shown to be more sensitive, convenient, and objective than traditional cytopathic effect based methods (Aste-Amezaga et al., 2004; Zheng et al., 2017), and thus enabled the surveillance of pre-exiting anti-HAdV4 nAb and anti-HAdV7 nAb in large cohorts. To our knowledge, we are the first to report the seroprevalence of nAb to HAdV4 and HAdV7 in civilian population. We found that, the seropositivity rates of anti-HAdV4 nAb and anti-HAdV7 nAb were 58.4 and 63.8% (Figure 2), respectively, which were lower than that of anti-HAdV5 nAb but higher than that of anti-HAdV14 nAb and anti-HAdV55 nAb (Hong et al., 2001; Sun et al., 2011; Zheng et al., 2017). At least 41.6 and 36.2% of individuals in this population are still susceptible for HAdV4 and HAdV7 infection respectively, due to the absence of protective nAb (Barraza et al., 1999; Clemmons et al., 2017). Our results reinforced that HAdV4 and HAdV7 should be considered when ARD outbreaks. A prophylactic vaccine may be necessary for the prevention of HAdV4 and HAdV7 infection.

Recently, the pre-existing nAb to a variety of HAdVs including HAdV2, 5, 14, 26, 35, and 55 have been investigated (Barouch et al., 2004; Abbink et al., 2007; Zheng et al., 2017). However, Investigations of anti-HAdV4 nAb and anti-HAdV7 nAb were mainly conducted in military recruits (Ludwig et al., 1998; Kolavic-Gray et al., 2002; Choudhry et al., 2016). Results from United States military trainees revealed that the overall seroprevalence of anti-HAdV4 nAb was significantly lower than that of anti-HAdV7 nAb, whereas individuals with high titers of anti-HAdV4 nAb were much more than those with high titers of anti-HAdV7 nAb (Ludwig et al., 1998). Our results in civilian populations showed a similar trend. That is, the frequency of HAdV4-seropositive individuals is lower than that of HAdV7-seropositive ones, but the titers of anti-HAdV4 nAb were significantly higher than that of anti-HAdV7 nAb (Figure 2). The similar results from two different populations imply that HAdV4 circulation in these populations may be more recent than HAdV7, or HAdV4 infection may induce stronger nAb responses than HAdV7 infection.

Several studies have shown that the seroprevalence of nAb to other HAdVs such as HAdV5 increased with age (Sun et al., 2011; Yu et al., 2012), but the nAb titers would decrease gradually in years post infection due to cumulative saturation of infection in adults (Yang et al., 2016). However, we found that both HAdV4 and HAdV7 seropositivity rates gradually increased with age (Figure 3), consistent to previous results from army trainees (Ludwig et al., 1998), suggesting that infection rates of HAdV4 and HAdV7 would not be saturated in adults. The sporadic appearance of adult patients with HAdV4- or HAdV7-associated severe pneumonia also supported the absence of protective immunity in a proportion of individuals (Barraza et al., 1999; Clemmons et al., 2017). Thus, close attention should be paid to HAdV4- and HAdV7-associated ARDs not only for children, but also for adults. Similar to HAdV14 and HAdV55 seroprevalence (Zheng et al., 2017), sex is not an important factor influencing either seropositivity rates or nAb titers of HAdV4 and HAdV7 (Figure 4). Intriguingly, we found that HAdV4-seropositive individuals tend to be HAdV7-seropositive, and *vice versa* (Figure 5). Given that HAdV4 antisera and HAdV7 antisera showed no significant cross-neutralizing activity to HAdV4 and HAdV7 (Figure 1), this result implied that a proportion of individuals might be more susceptible to HAdV infection. These individuals may be successively or simultaneously infected by HAdV4 and HAdV7 and thereby generated nAb responses to both of them. However, the mechanisms underlying this phenomenon need to be elucidated in future studies.

Recombinant adenoviral vectors based on HAdV4 and HAdV7 have been explored as vaccine vectors for a variety of pathogenic viruses (Hung et al., 1990; Hsu et al., 1992; Alexander et al., 2012; Weaver, 2014; Khurana et al., 2015). A potential limitation of recombinant adenoviral vectors is the pre-existing nAb resulted from natural exposure (Lanzi et al., 2011). The sensitive neutralization assays established in this study can potentially facilitate the screening of HAdV4- and HAdV7-seronegative individuals, which may be important for the clinical evaluation of vaccines based on recombinant HAdV4 or HAdV7 vectors. In this particular cohort, 41.6 and 36.2% of participants were seronegative for anti-HAdV4 nAb and anti-HAdV7 nAb, respectively, and thereby could be included in the clinical trials for HAdV4- or HAdV7-vectored vaccines.

HAdV4 has been shown to originate from a recombinant event between HAdV16 and simian adenovirus type 26 (SAdV26) (Dehghan et al., 2013), and comprises loop 1 and loop 2 of HAdV16 hexon in the genome chassis of SAdV26. Because hexon loop 1 and loop2 contain the major targets of anti-HAdV nAb (Pichla-Gollon et al., 2007; Yuan et al., 2009; Feng et al., 2018), screening of anti-HAdV4 nAb with HAdV4-SEAP cannot discriminate the nAb induced by HAdV16 infection. However, dissecting the seroprevalence of anti-HAdV4 nAb, resulted no matter from HAdV4 infection or from HAdV16 infection, could help to understand the herd immunity to HAdV4. Moreover, to our best knowledge, no outbreaks of HAdV16 infection have ever been reported in Hunan province, China. The circulation of HAdV16 in this district is not dominant and even rare. Therefore, the pre-existing anti-HAdV4 nAb in the investigated cohorts was most likely resulted from HAdV4 infection but not HAdV16 infection. Future studies, such as detection of fiber-specific nAb and surveillance of the circulating HAdV strains may help to clarify the real seroprevalence of anti-HAdV4 nAb induced by HAdV4 infection.

In summary, we developed reporter virus based neutralization assays for HAdV4 and HAdV7, and investigated the pre-existing nAb responses in a civilian population. HAdV4 and HAdV7 can be potential causative pathogens for ARDs in civilian populations in southern China. Our results provided insightful information not only for understanding the herd immunity to these two HAdVs but also for the development of vaccines against HAdV infection.

## Data Availability Statement

The raw data supporting the conclusions of this manuscript will be made available by the authors, without undue reservation, to any qualified researcher.

## Author Contributions

XY, LX, and XZ collected the samples, performed the neutralization assays and analyzed the results. JW, TS, YF, XL, WS, and QW contributed to the data analysis. CL reviewed the manuscript. LC and LF conceived and designed the study, wrote the manuscript, and approved the submission.

## Conflict of Interest Statement

The authors declare that the research was conducted in the absence of any commercial or financial relationships that could be construed as a potential conflict of interest.
