# Supplementary material for: Seroprevalence of Neutralizing Antibodies to Human Adenovirus Type 4 and 7 in Healthy Populations From Southern China
Source: Front Microbiol. 2018 Dec 10;9:3040. doi: 10.3389/fmicb.2018.03040 (PMC6295555; doi:10.3389/fmicb.2018.03040)
Supplement: Supplementary file 1 [file Table_1.docx]

Supplementary Material

Seroprevalence of neutralizing antibodies to human adenovirus type 4 and 7 in healthy populations from Southern China

Xianmiao Ye^1,2,†^, Lijun Xiao^3,†^, Xuehua Zheng^1,†^, Jinlin Wang^1,2^, Tao Shu^1,2^, Ying Feng^4^, Xinglong Liu^1^, Wan Su^1,5^, Qian Wang^1,2^, Chufang Li^4^, Ling Chen^1,4,^*, and Liqiang Feng^1,^*

^1^ State Key Laboratories of Respiratory Diseases, Guangzhou Institutes of Biomedicine and Health, Chinese Academy of Sciences, Guangzhou, China, ^2^ University of Chinese Academy of Sciences, Beijing, China, ^3^ Center for Disease Control and Prevention of Chenzhou, Chenzhou, China, ^4^ The First Affiliated Hospital of Guangzhou Medical University, Guangzhou, China, ^5^ School of Biomedical Sciences, Huaqiao University, Quanzhou, China

*** Correspondence:** Liqiang Feng, [feng_liqiang@gibh.ac.cn](mailto:feng_liqiang@gibh.ac.cn); Ling Chen, [chen_ling@gibh.ac.cn](mailto:chen_ling@gibh.ac.cn)

**Supplementary Table S1. Demographics of serum sample donors.**

| **Groups** | **N (%)** |
| --- | --- |
| **Age (years)** |  |
| ≤20 | 200(15.4%) |
| 21-30 | 294(22.6%) |
| 31-40 | 286(22.0%) |
| 41-50 | 305(23.4%) |
| >50 | 203(15.6%) |
| Unknown^a^ | 14(1.1%) |
| **Sex** |  |
| Male | 704(54.1%) |
| Female | 598(45.9%) |

^a^ Age information of 14 donors was unknown.

**Supplementary Table S2. Seroprevalence of anti-HAdV4 nAb in the cohort.**

|  | **anti-HAdV4 nAb titers**  **N(%)** | | | | **Total (Positive%)** |
| --- | --- | --- | --- | --- | --- |
|  | **<72** | **72-200** | **201-1000** | **>1000** | **≥72** |
| **Age(years)** |  |  |  |  |  |
| ≤20 | 131(65.5) | 5(2.5) | 10(5.0) | 54(27.0) | 69(34.5) |
| 21-30 | 139(47.3) | 27(9.2) | 41(13.9) | 87(29.6) | 155(52.7) |
| 31-40 | 115(40.2) | 27(9.4) | 52(18.2) | 92(32.2) | 171(59.8) |
| 41-50 | 105(34.4) | 32(10.5) | 58(19.0) | 110(36.1) | 200(65.6) |
| >50 | 48(23.6) | 13(6.4) | 46(22.7) | 96(47.3) | 155(76.4) |
| Unknown^a^ | 4(28.6) | 1(7.1) | 5(35.7) | 4(28.6) | 10(71.4) |
| Total | 542(41.6) | 105(8.1) | 212(16.3) | 443(34.0) | 760(58.4) |
| **Sex** |  |  |  |  |  |
| Male | 280(39.8) | 57(8.1) | 114(16.2) | 253(35.9) | 424(60.2) |
| Female | 262(43.8) | 48(8.0) | 98(16.4) | 190(31.8) | 336(56.2) |
| Total | 542(41.6) | 105(8.1) | 212(16.3) | 443(34.0) | 760(58.4) |

^a^ Age information of 14 people was unknown

**Supplementary Table S3. Seroprevalence of anti-HAdV7 nAb in the cohort.**

|  | **anti-HAdV7 nAb titers**  **N(%)** | | | | **Total(Positive%)** |
| --- | --- | --- | --- | --- | --- |
|  | **<72** | **72-200** | **201-1000** | **>1000** | **≥72** |
| **Age(years)** |  |  |  |  |  |
| ≤20 | 133(66.5) | 24(12.0) | 24(12.0) | 19(9.5) | 67(33.5) |
| 21-30 | 123(41.8) | 41(13.9) | 72(24.5) | 58(19.7) | 171(58.2) |
| 31-40 | 92(32.2) | 43(15.0) | 75(26.2) | 76(26.6) | 194(67.8) |
| 41-50 | 82(26.9) | 49(16.1) | 93(30.5) | 81(26.6) | 223(73.1) |
| >50 | 36(17.7) | 24(11.8) | 82(40.4) | 61(30.0) | 167(82.3) |
| Unknown^a^ | 5(35.7) | 3(21.4) | 3(21.4) | 3(21.4) | 9(64.3) |
| Total | 471(36.2) | 184(14.1) | 349(26.8) | 298(22.9) | 831(63.8) |
| **Sex** |  |  |  |  |  |
| Male | 261(37.1) | 97(13.8) | 192(27.3) | 154(21.9) | 443(62.9) |
| Female | 210(35.1) | 87(14.5) | 157(26.3) | 144(24.1) | 388(64.9) |
| Total | 471(36.2) | 184(14.1) | 349(26.8) | 298(22.9) | 831(63.8) |

^a^ Age information of 14 people was unknown
